# Robotic-Assisted Laparoscopic Prostatectomy for High-Risk Prostate Cancer: Technical Considerations and Review of the Literature

**DOI:** 10.5402/2011/201408

**Published:** 2011-09-25

**Authors:** Sean P. Stroup, Christopher J. Kane

**Affiliations:** ^1^Division of Urology, University of California, 200 West Arbor Drive 8897, San Diego, CA 92103-8897, USA; ^2^Section of Urologic Oncology, Moores UCSD Cancer Center, La Jolla, CA 92093, USA; ^3^Urology Section Veterans Affairs San Diego Medical Center, La Jolla, CA 92103, USA

## Abstract

Men with high-risk prostate cancer are at significant risk of progressive, symptomatic disease leading to metastases or death from prostate cancer. Surgery—specifically robotic-assisted laparoscopic prostatectomy (RALP)—is increasingly being considered as a key component of a multimodal strategy to treat these patients. Herein, we review key technical considerations of performing RALP with bilateral pelvic lymphadenectomy in men with high-risk disease. Recent literature supporting the increasing role of surgery either alone or in combination with adjuvant therapies to treat men with high-risk prostate cancer is also reviewed.

## 1. Introduction

Men with localized high-risk prostate cancer represent a group at significant risk for metastases and death from prostate cancer [1]. Historically, surgery was not the preferred option due to concerns about adequately assessing subclinical metastatic disease, increased risk of positive surgical margins, and a lack of randomized trials showing a significant clinical benefit of radical prostatectomy over radiotherapy [2, 3]. In the current era, where fewer men present with bulky disease and most are considered high-risk by Gleason grade alone, radical prostatectomy with pelvic lymph node dissection (PLND) has become an important and effective treatment option for men with localized high-risk disease [4]. In addition, with the diffusion of robotic-assisted laparoscopic prostatectomy (RALP) into the urologic community, this technique is being increasingly adopted to treat high-risk patients [5–7]. Although there are several technical considerations, in experienced hands RALP can maintain oncological principles while minimizing patient morbidity. Herein we review the increasing role of RALP with pelvic lymphadenectomy either alone or in combination with adjuvant therapies as a key component of a multimodal treatment strategy for men with high-risk prostate cancer. Ultimately, successful treatment approaches require careful risk stratification, delivery of quality treatment with curative intent, consideration of multimodal approaches, and management of patient expectations within a therapeutic context. 

## 2. Defining High-Risk Prostate Cancer

The characteristics of men diagnosed with prostate cancer have changed dramatically over the last 2 decades largely as a result of widespread serum PSA testing. Men with prostate cancer now present with lower clinical stage and serum PSA, and a greater proportion have low-risk features [8]. 

The characteristics of patients with high-risk prostate cancer are also changing. Fewer patients are now diagnosed with advanced prostate cancer than early in the PSA era. Historically, nearly 40% of men presented with high-risk disease based on clinical stage. Now only 15% of men present with high-risk disease as a result of this stage migration [4]. Nearly 60% of high-risk cases in the current era are characterized as such based on Gleason grading alone. Fundamental work by Pound and colleagues helped to establish the natural history of prostate cancer in surgically treated patients [9]. At a median of 8 years they identified biochemical recurrence in 15% and found metastatic disease developing in 34% of this group. In survival analysis, time to biochemical progression (*P* < .001), Gleason score (*P* < .001), and PSA doubling time (*P* < .001) were predictive of the probability and time to the development of metastatic disease.

D'Amico et al. continued this work by refining criteria to identify those at high risk of progression. They identified clinicopathological features that predicted adverse outcomes, including development of metastatic disease and death from prostate cancer, and in doing so, helped to establish the most commonly accepted definition [10]. They identified PSA > 20 ng/mL, Gleason grade 8–10, and >cT2c findings on digital rectal examination as important factors to identify men at the highest risk of progression.

## 3. Optimal Treatment

There is no consensus on the optimal treatment of high-risk nonmetastatic prostate cancer. Historically, prostatectomy for Gleason 8–10 disease was not considered curative, and many practitioners directed patients towards external beam radiotherapy or primary hormonal treatment. Cooperberg et al. identified significant differences in practice patterns among urologists for treatment of men with high-risk disease. In their study spanning from 1998 to 2007, men with high-risk disease were offered primary androgen deprivation therapy or external beam radiotherapy over radical prostatectomy [11]. Other studies show that men with high-grade prostate cancer may be cured with surgery either alone or in combination with radiotherapy ± androgen deprivation therapy [12–15]. The Shared Equal Access Regional Cancer Hospital (SEARCH) group has also shown that not all men with biopsy Gleason 7–10 disease have poor outcomes [16]. In this study, men with pathologic Gleason 4 + 3 experienced similar rates of biochemical recurrence as those with Gleason 8–10 cancer. Based on these findings radical prostatectomy has become a key component of multimodal therapy for men with high-risk disease [17]. 

## 4. Staging and Risk Assessment of High-Grade Prostate Cancer

### 4.1. Clinical Features: Gleason Score, PSA, and DRE

Not all high-risk prostate cancers are defined by a single parameter, but Gleason score, PSA, PSA kinetics, and digital rectal exam used in combination are useful in assigning risk and clinically staging patients. Gleason biopsy grading is one of the strongest predictors of biochemical recurrence (BCR) as well as disease-specific and overall survival [9, 10, 18]. In general, the more granular the pathologic assessment of biopsy material, the better risk assessment can be made. That is, the number, percentage, or total length of cancer detected per number of cores or amount of tissue sampled provides important diagnostic information to the clinician for risk stratification. These details are also increasingly being incorporated into risk stratification models such as the Kattan, CPDR, and CAPRA nomograms [19, 20].

In the current era few patients are characterized as high risk by PSA alone. Although elevated PSA levels may be due to cancer, benign prostatic enlargement and inflammatory or infections conditions may also increase concentrations. In these patients, evaluation of metastatic disease takes on greater importance and may include bone scan and computed tomography of the abdomen and pelvis. Interesting work in the area of red bone marrow axial skeleton magnetic resonance imaging has also demonstrated improved sensitivity and specificity at detecting metastasis from prostate cancer [21, 22].

Clinically advanced disease with digital exam findings of cT3 or greater are less common now than in the past. In these patients advanced imaging techniques including endorectal coil MRI or detailed ultrasound examinations of the prostate with or without power Doppler may help better define the lesion [23]. Identification of bladder neck or seminal vesicle invasion can be used to help surgical planning.

### 4.2. Staging and Treatment Considerations

Age, comorbidities, and life expectancy should be considered before deciding on a treatment strategy, as not all options may be reasonable for every patient. Surgery offers patients the potential of long-term cure with the distinct advantage of local disease control. Prostatectomy has been shown to prevent local complications including recurrent hematuria, urinary retention, pain and hydronephrosis. Careful discussion of expectations and outcomes is necessary before considering local therapy with curative intent. Although many patients with high-risk prostate cancer expect cure, prostatectomy can only achieve this for some 40%–60% of patients. Many will need to consider surgery as only one arm in a multimodal approach.

## 5. Technique of Robotic-Assisted Laparoscopic Prostatectomy in High-Risk Prostate Cancer

### 5.1. Approach and Access

The majority of RALP procedures are performed using the transperitoneal approach. In addition to ease of access, this technique offers advantages of a large working space with better proximal and medial access to lymph nodes over a retroperitoneal approach. Several technical considerations may facilitate RALP in patients with high-risk disease (Table 1).

The robot is docked after establishing pneumoperitoneum and placing ports. Typical port placement includes an 8 mm robotic port 15 cm lateral to the umbilicus on the right; a single 12 mm assist port 6 cm lateral to the umbilicus on the right; two left-sided 8 mm robotic ports, one 10 cm lateral to the umbilicus, and one 3 cm medial to the anterosuperior iliac spine. Close inspection of the abdomen will identify any initial access injuries and bowel adhesions that must be divided before proceeding. We favor a posterior approach with initial reflection of the sigmoid colon to gain a better view of the pelvis. 

Next we place gentle cranial retraction on the rectum and make a transverse peritoneotomy between the bladder and the rectum. After transaction of each vas deferens, the seminal vesicles are dissected circumferentially and 5-mm polymer clips are placed on the small blood vessels that emanate from their tips. High-risk prostate cancer may be associated with firm seminal vesicle tissue that is characteristic of invasion and requires careful robotic manipulation to ensure complete removal. While lifting the seminal vesicles, a transverse incision is made in Denonvillier's fascia to establish the dissection plane between the rectum and prostate. Establishing this plane may be difficult in case of advanced disease, prior treatment with androgen ablation or radiotherapy, and in the setting of multiple prior biopsies or transurethral resection of the prostate; however, this is not universal.

### 5.2. Endopelvic Dissection

The anterior dissection begins by opening the space of Retzius by dividing from one medial umbilical ligament to the other and the urachus in the midline. The peritoneum is opened widely down to where the vas deferens crosses the external iliac vein. Firm cranial retraction on the bladder facilitates evaluation of the anterior surface of the prostate. High release of endopelvic fascia is initiated with sharp dissection, and the dorsal venous complex is controlled with an endo-GIA stapler using a green load.

### 5.3. Nerve Sparing

The degree of nerve sparing should be determined by exam, clinical risk stratification, and preoperative imaging if available. Several authors have demonstrated nerve-sparing RALP can be safely performed in patients with preoperatively high-risk prostate cancer [5]. In a recent report, Shikanov and colleagues evaluated potency and positive surgical margins after intrafascial or extrafascial nerve sparing [24]. They found a trend toward lower positive surgical margins in the pT3 group (51% versus 28%; *P* = 0.08). Further, they noted significantly fewer mid- and posterolateral positive margins in the extrafascial group 11% versus 37% and 11% versus 29%, respectively (*P* < 0.001), at the expense of slightly worse potency at 12 months postoperatively.

### 5.4. Bladder Neck Dissection

The anterior bladder neck is then incised with electrocautery. With the Foley catheter now lifted, the prostate is circumferentially dissected from the base of the prostate. After opening clearly entering the retrovesical space the previously dissected seminal vesicles may be elevated to assist with pedicle ligation. Urologists should avoid the natural tendency to advance towards the prostate and use a perpendicular place of dissection between the bladder neck and prostate base to avoid a positive surgical margin. Frozen section analysis may be useful in selected cases [36, 37]. Visual clues to decrease the risk of posterior-lateral surgical margins include appreciation of periprostatic (lateral prostatic) fascial compartments; color and texture of the tissue; periprostatic veins as a landmark for athermal dissection; signs of inflammation; a freely separating bloodless plane showing loose shiny areolar tissue [38].

Athermic technique with use of surgical clips may be used to complete pedicle ligation and mobilization of the prostate base. Meticulous circumferential dissection of the prostate apex is necessary to avoid positive surgical margins near the urethra. The vesicoureteral anastamosis is completed after pelvic lymphadenectomy using a running 3–0 double-armed, absorbable suture with care taken to avoid inclusion of rhabdosphincter fibers in the anastamosis. The tendency to perform a wide dissection here is also a potential for complications. Rectal injury is more common in the setting of retrograde dissection with an inadequately dissected posterior plane. A wide dissection also threatens urinary continence by sacrificing both urethral length and rhabdosphincter integrity.

## 6. Review of Robotic Assisted Laparoscopic Prostatectomy Series for High-Risk Disease

The proportion of men undergoing robotic prostatectomy diagnosed with high-risk disease is increasing. This trend has been noticed at large academic centers and will likely to continue as more low-risk patients select active surveillance [39]. Several RALP series focused on high-risk prostate cancer have been published (Table 2). Within this group there is significant variation in the definition of high risk, which makes comparison across studies challenging. 

Locally advanced prostate cancer characterized by clinical T3 or greater disease may be among the most difficult challenges for RALP. Bulky disease and increased risk of bladder neck involvement or seminal vesicle invasion can make robotic dissection difficult. Casey et al. demonstrated excellent short-term results in 35 patients with pathologic T3 disease who underwent RALP. At a median of 13 months followup, they noted 28.6% with biochemical recurrence, 37% with seminal vesicle invasion, and 20% with positive margins. 

RALP has also been studied in other high-risk, organ-confined prostate cancer groups. Men with one or more other high-risk features including PSA ≥ 20 ng/mL and Gleason score 8–10 are included here. Surgical skill and technical expertise drive outcomes particularly in these cases. Inadvertent capsular incisions that create positive margins, excessive neurovascular bundle resection, and poor management of urethral length are among the most important potentially avoidable surgical errors.

## 7. Pelvic Lymphadenectomy

The role and extent of pelvic lymphadenectomy at the time of radical prostatectomy have evolved in recent history. There is ongoing discussion in the literature regarding who should undergo pelvic lymph node dissection (PLND), the extent of the dissection that should be performed and whether PLND provides therapeutic benefit in addition to staging information. In patients with high-risk prostate cancer we perform a bilateral extended pelvic lymph node dissection before completion of the vesicoureteral anastamosis [40]. Investigators have clearly shown that the lymphatic drainage of the prostate is not limited to the obturator and external iliac lymph nodes, and thus a PLND limited to these regions does not address all the potential draining sites. An extended pelvic lymph node dissection (EPLND), including the internal iliac lymph nodes along with external iliac and obturator nodes, more accurately reflects the true lymphatic drainage of the prostate, increases nodal yield, and improves detection of metastatic lymph nodes.

An emerging body of evidence supports completion prostatectomy in the setting of positive lymph nodes to provide local control. In a carefully matched group of patients with lymph node metastasis at the Mayo Clinic, the 10-year probability of overall survival was 66% for those undergoing prostatectomy with subsequent androgen deprivation therapy and 28% for those treated with androgen deprivation alone [41]. In a study from Germany, Engel et al. published results also supporting a survival advantage in those treated with prostatectomy in the setting of positive pelvic lymph nodes [42]. 

### 7.1. Robotic Extended Pelvic Lymph Node Technique

Robotic EPLND begins by carefully incising the peritoneum overlying the common iliac artery, taking care to remain lateral to the ureter. Medial retraction of the bladder can facilitate identification of triangular apex of lymphatic tissue that sits at the bifurcation of the common iliac artery. The dissection of lymphatic tissue is antegrade down along the hypogastric artery to the origin of the medial umbilical ligament. These internal iliac (hypogastric) nodes are omitted in the standard PLND. Next we dissect the lymphatic tissue along the external iliac artery down to the node of Cloquet. The crossing circumflex vein can often be preserved with careful dissection. Retrograde dissection along the external iliac vein and sweeping maneuvers towards the elevated packet facilitate separation of the obturator nodes. Medial and lateral nodal tissue may be split over the obturator nerve to facilitate the dissection. External iliac, obturator, and internal iliac nodes can often be removed en block. Surgeons have used mono or bipolar electrocautery, polymer clips, or metal clips to ligate vessels and lymphatics. All appear to have similar complication rates. Select placement of metal clips at the bladder pedicles and in the area of lymphadenectomy may facilitate postoperative radiotherapy. A committed surgical approach that respects anatomic pitfalls can yield excellent nodal counts while minimizing complications.

## 8. Review of Robotic-Assisted Extended Pelvic Lymph Node Dissection

Pelvic lymphadenectomy during RALP is technically feasible and appears to have minimal morbidity as well as high lymph node yield which may improve pathologic staging and provide therapeutic benefit.  Table 3 summarizes current extended and standard lymphadenectomy robotic series, including all risk categories. Several studies note that men with limited micrometastatic disease may be cured by PLND at the time of radical prostatectomy. In one series, Yee et al. reported on the feasibility of robotic assisted extended pelvic lymph node dissection in 32 men [30]. In their study EPLND included obturator, internal iliac, external iliac, and common iliac nodes to bifurcation of aorta. Positive lymph nodes were noted in 12.5% after a median of 18 nodes removed per patient, and there were no lymphoceles or serious complications. A report by Feike et al. identified lymph node metastases in 16% of patients with a mean 19.4 nodes resected. Twenty-five percent of positive nodes were detected in the internal iliac distribution. 

## 9. Integrating Additional Therapy

### 9.1. Adjuvant and Salvage Radiotherapy

Three randomized controlled trials are available that have addressed the timing or need for adjuvant external beam radiotherapy after prostatectomy: the German Cancer Society trial, the EORTC 22911, and the SWOG 8794 trial. All have supported adjuvant external beam radiotherapy after prostatectomy for advanced localized prostate cancer by demonstrating improved biochemical recurrence-free survival rates and improved metastasis-free survival in the SWOG trial. Those with positive surgical margins and locally advanced disease are most likely benefit from adjuvant radiotherapy. Adjuvant radiotherapy is generally administered between 3 and 6 months postoperatively. It is unclear what differences exist between early salvage radiotherapy and adjuvant radiotherapy in the era of superselective PSA. Generally patients experience only modest toxicity.

## 10. Salvage RALP in Men with High-Risk Features

Recurrence in high-risk disease results from either inadequate primary treatment or interval development metastatic disease. Salvage prostatectomy is generally reserved for men with evidence of local disease. Careful restaging and metastatic workup with repeat prostate biopsy, abdomen-pelvic imaging, and consideration of bone scan or axial skeleton bone marrow MRI are requisite. A thorough discussion with patients is needed with particular emphasis on postoperative functional outcomes, including continence and erectile function. 

## 11. Conclusion

RALP with pelvic lymphadenectomy either alone or in combination with adjuvant therapy is an important treatment option for men with high-risk prostate cancer. RALP can provide effective management of locally advanced and high-risk prostate cancer while achieving excellent oncological outcomes. Surgical experience and close attention to preservation of anatomic structures may improve outcomes. Extended pelvic lymph node dissection plays an important therapeutic and staging role in management of high-risk prostate cancer.

## Figures and Tables

**Table 1:** Technical considerations for RALP with extended pelvic lymph node dissection in high-risk patients.

Division of lateral physiologic adhesions of rectum and sigmoid to left pelvic side wall, facilitating elevation of bowel out of pelvis.	
Posterior approach begins with perneotomy and dissection of seminal vesicles under direct vision.	
Incise Denonvillier's fascia under elevated seminal vesicles and establish safe plane between prostate and rectum.	
Mobilize bladder and incise peritoneum to level of vas deferens bilaterally to facilitate extended pelvic lymph node dissection.	
Consider extrafascial or modified nerve sparing with medial endopelvic fascia incision to balance oncologic control with quality of life outcomes.	
Err towards bladder while opening anterior bladder neck.	
Meticulous circumferential dissection of the prostate apex is necessary to avoid positive surgical margins.	
Identify ureter crossing over common iliac artery and incise peritoneum to begin extended pelvic lymph node dissection.	
Consider placement of metal clips at prostate pedicles and during lymphadenectomy to facilitate targeting of postoperative radiotherapy.	

**Table 2:** Published robotic assisted laparoscopic prostatectomy series for high risk disease.

Series	Patients	Risk group	Nodes + (%)	Seminal vesicle invasion (%)	Positive margin (%)	Biochemical recurrence (%)/median followup	Adjuvant therapy (%)
Lavery et al. 2010 [5]	123	D'Amico high risk*	2.4	32	31	26	26
Ham et al. [6]	121	≥cT3a locally advanced	24	—	48.8	—	—
Shikanov et al. [7]	70	Biopsy Gleason 8–10	12.9	14	24.2	13/9.6 mo.	13
Casey et al. [25]	35	Final ≥pT3, with 29% D'Amico high risk*	19	37	20	28.6/13 mo.	37
Jayram et al. [26]	148	D'Amico high risk*	12.3		20.5	21.3/24 mo.	23.3
Yee et al. [27]	62	D'Amico high risk*	—	—	22.6	—	
Badani et al. [28]	177	D'Amico high risk*	—	—	35^†^	47.2/22 mo.^‡^	

*D'Amico's criteria for high-risk prostate cancer were utilized: prostate-specific antigen ≥20 ng/mL, clinical stage ≥T2c, or preoperative Gleason grade ≥8.

^†^Assessed in patients with pathologic T3 disease.

^‡^Assessed in patients with pathologic Gleason grade ≥8.

**Table 3:** Summary of robotic extended and standard pelvic lymph node dissection series for RALP.

Robotic series	Patients	Mean lymph nodes retrieved (range)	Patients with positive lymph nodes (%)	Clinical lymphocele (%)
Extended				
Feicke et al. [29]	99	19	16	4
Yee et al. [30]	32	18	12.5	0
Yates et al. [31]	62	3.3	3.2	—
Truesdale et al. [32]	99	6	1	—
Standard				
Atug et al. [33]	40	14.1	5	0
Polcari et al. [34]	60	8.2	3.3	3
Zorn et al. [35]	296	12.5	7.7	2
